# Paradigm shifts in genomics through the FANTOM projects

**DOI:** 10.1007/s00335-015-9593-8

**Published:** 2015-08-08

**Authors:** Michiel de Hoon, Jay W. Shin, Piero Carninci

**Affiliations:** Division of Genomic Technologies, RIKEN Center for Life Science Technologies, Yokohama, 230-0045 Japan

## Abstract

Big leaps in science happen when scientists from different backgrounds interact. In the past 15 years, the FANTOM Consortium has brought together scientists from different fields to analyze and interpret genomic data produced with novel technologies, including mouse full-length cDNAs and, more recently, expression profiling at single-nucleotide resolution by cap-analysis gene expression. The FANTOM Consortium has provided the most comprehensive mouse cDNA collection for functional studies and extensive maps of the human and mouse transcriptome comprising promoters, enhancers, as well as the network of their regulatory interactions. More importantly, serendipitous observations of the FANTOM dataset led us to realize that the mammalian genome is pervasively transcribed, even from retrotransposon elements, which were previously considered junk DNA. The majority of products from the mammalian genome are long non-coding RNAs (lncRNAs), including sense-antisense, intergenic, and enhancer RNAs. While the biological function has been elucidated for some lncRNAs, more than 98 % of them remain without a known function. We argue that large-scale studies are urgently needed to address the functional role of lncRNAs.

## Pre-FANTOM Era

In the 1990’s, mouse genetics was considered a powerful tool to discover genes responsible for phenotypes and ultimately to model function of disease-related genes. However, such gene hunting was based on positional cloning, which required laborious work of a skilled researcher to identify causative genes of a given phenotype (Collins 1995). Accelerating this process required the development of genetic maps as well as a complete set of transcribed genes to overlay these DNA maps. Realizing the need of the community for a comprehensive description of the mammalian genome, RIKEN decided to enter and contribute to the emerging area of genomics by focusing on the transcriptome rather than on genome sequencing. Thus, in 1995 RIKEN embarked on the mouse genome encyclopedia project with the aim to produce the broadest collection of full-length cDNA. This also generated a resource for future experiments, such as the expression of cDNAs for functional studies (Shin et al. 2012; Suzuki et al. 2012), structure determination of the proteins (Kasai et al. 2004), and applied sciences such as induced pluripotent stem (iPS) cells (The Bungeishunju 2010; Newton 2012).

For this project to succeed, it was crucial to develop a series of experimental protocols to efficiently reverse-transcribe the complete transcript, generating full-length cDNAs. A particular problem was that the reverse transcriptase tends to dissociate from the transcript when it encounters secondary structures in the RNA, which resulted in truncated cDNAs. Simply increasing the temperature of the reverse transcription reaction to dissolve such secondary structures led to inactivation of the reverse transcriptase. We overcame this challenge by establishing a reverse transcription protocol using trehalose to maintain the enzymatic activity of the reverse transcriptase at higher temperatures (Carninci et al. 1998, 2002). Complementary to this methodology, we further developed (1) The cap-trapper method to select for full-length cDNAs while avoiding truncated ones (Carninci et al. 1996, 1997), (2) a vector system able to host long cDNA clones that can shuttle them from a lambda phage to a plasmid without size or sequence bias (Carninci et al. 2001), and (3) methods to ensure the inclusion of the very first transcribed nucleotide (Shibata et al. 2001).

In parallel to establishing the experimental protocols, we also developed the RIKEN integrated sequence analysis (RISA) 384 multicapillary sequencer—the only capillary sequencing instrument able to sequence 384 cDNAs in parallel (Shibata et al. 2000, 2001)—as well as the plasmid extractor instrument (Itoh et al. 1999), which was used to generate and sequence 40,000 clones per day to screen for novel cDNAs.

However, in our quest to build the broadest cDNA collection, we were faced with a new challenge: cDNA libraries resulted in sequencing the same highly expressed transcripts repeatedly while missing lowly expressed transcripts. It therefore became essential to find a way to enrich for lowly expressed and novel transcripts in the cDNA libraries while avoiding cDNAs that had already been sequenced. For this purpose we devised a method to normalize and subtract full-length cDNA libraries (Carninci et al. 2000), which was later expanded into a reiterative subtraction strategy using even a small amount of cDNA (Hirozane-Kishikawa et al. 2003). After sequencing a cDNA library, we rearrayed the novel cDNAs and used them to produce an RNA driver that was employed to remove these cDNAs for the next series of libraries. Reiteration of this process, with a growing pool of cDNA used as driver, allowed us to progressively discover novel RNAs, including rare transcripts (Carninci et al. 2003). In total, we produced 246 normalized, subtracted cDNA libraries from a wide collection of mouse tissues and cells, which were ideal as it also allowed sampling of developmental stages and rare tissues, including tiny nervous tissues, to ensure the largest coverage possible.

We started sequencing the initial libraries in 1998, and by 1999, the collection was already rich with a large number of ESTs as well as full-length cDNAs. However, while the project continued as a huge technical success for making and sequencing cDNAs, the bioinformatics analysis was not well considered at that time. For most novel cDNA sequences, BLAST against GenBank resulted in the uninformative “similar to EST” output. Thus, we were discovering novel mammalian transcripts at an unprecedented rate but were unable to annotate them.

## FANTOM1: The first annotation of full-length cDNAs

To address this concern, we decided to contact Gerry Rubin, who had organized the first genome annotation effort, the Drosophila melanogaster “jamboree” (Adams et al. 2000). His advice convinced us of the need for an integrated annotation process involving both computational predictions as well as manual curation by scientists with expertise in specific aspects of biology. In a few months, we arranged the first Functional Annotation of the Mouse (later Mammalian) Genome (FANTOM1) consortium meeting in Tsukuba (Ibaraki, Japan; August 28–September 8, 2000), where we annotated the first set of ~20,000 cDNAs and developed bioinformatics tools for annotation. The FANTOM1 participants will remember the vibrant dynamism of the event, with night shifts of bioinformaticians creating useful annotation tools interspersed with long brainstorming sessions to decide which cDNAs should be annotated, as well as karaoke intervals to reset our minds for the next round of annotations.

By 2001, we accomplished the annotation of the first set of cDNAs (Kawai et al. 2001). In addition to their scientific value in and of themselves, mouse cDNAs were also instrumental in the identification of human coding genes and were used for the gene prediction of the human genome, which was published (Lander et al. 2001) 1 week after the FANTOM1 paper.

## FANTOM2: The expansion of the full-length cDNA collection

In parallel, our group continued the sequencing of full-length cDNAs. By the time of the publication of the first FANTOM manuscript, we had accumulated a much larger but still growing collection of cDNA sequences. This included the sequences that were derived from the subtracted cDNA libraries, thus containing rare transcripts. In the second FANTOM meeting in May 2002, we enlarged the annotation to a total of approximately 60,770 full-length cDNAs (Okazaki et al. 2002). The cDNA collection was used for the annotation of the mouse genome (Waterston et al. 2002), published back-to-back with our cDNA collection, thereby demonstrating the importance of full-length cDNA sequencing to annotate gene structure, initiation, termination, and splicing variants in mammalian genomes. Owing to the full-length cDNA subtraction technologies (Carninci et al. 2000), the collection started to reveal that there were unexpected transcripts, usually with lower abundance, which did not appear to code for proteins. They were later identified as antisense RNA and long non-coding RNAs (lncRNA).

A series of papers followed shortly after the release of FANTOM2 collection including the first analysis of sense-antisense transcription (Kiyosawa et al. 2003a, b) and lncRNA (Numata et al. 2003), together with multiple papers, published in a special issue of Genome Research, in which specific aspects of particular interest were analyzed covering various biological areas. This included the analysis of several major gene families that were for the first time broadly covered by full-length cDNAs (summarized in Okazaki and Hume 2003).

## FANTOM3: Discovering the world of non-coding RNA with CAGE

While we continued the sequencing of novel full-length cDNAs, we also foresaw that the strong polymorphisms in the 5’ end of sequenced transcripts would require the comprehensive mapping and quantification of transcription initiation sites (TSSs) in order to identify promoters and their usage in different tissues and cells; this would also help us to map transcriptional networks governing cell state and identity. We, therefore, started to develop a high-throughput method to map TSS that relied on concatenating short stretches of the 5’ end of full-length selected cDNAs. Sequencing concatenations of 10–20 of such sequence tags as single reads on the RISA 384 multicapillary sequencer allowed us to map TSSs at a reasonable cost. This was the first version of the CAGE (Cap-analysis gene expression) protocol (Shiraki et al. 2003), which has been further developed and improved continuously since its introduction. For example, oligo-(dT) priming was replaced by random priming to enable the inclusion of transcripts without poly-(A) tails (e.g., transcripts encoding histones, lncRNAs) in the reverse transcription reaction (Kodzius et al. 2006).

**Fig. 1:**
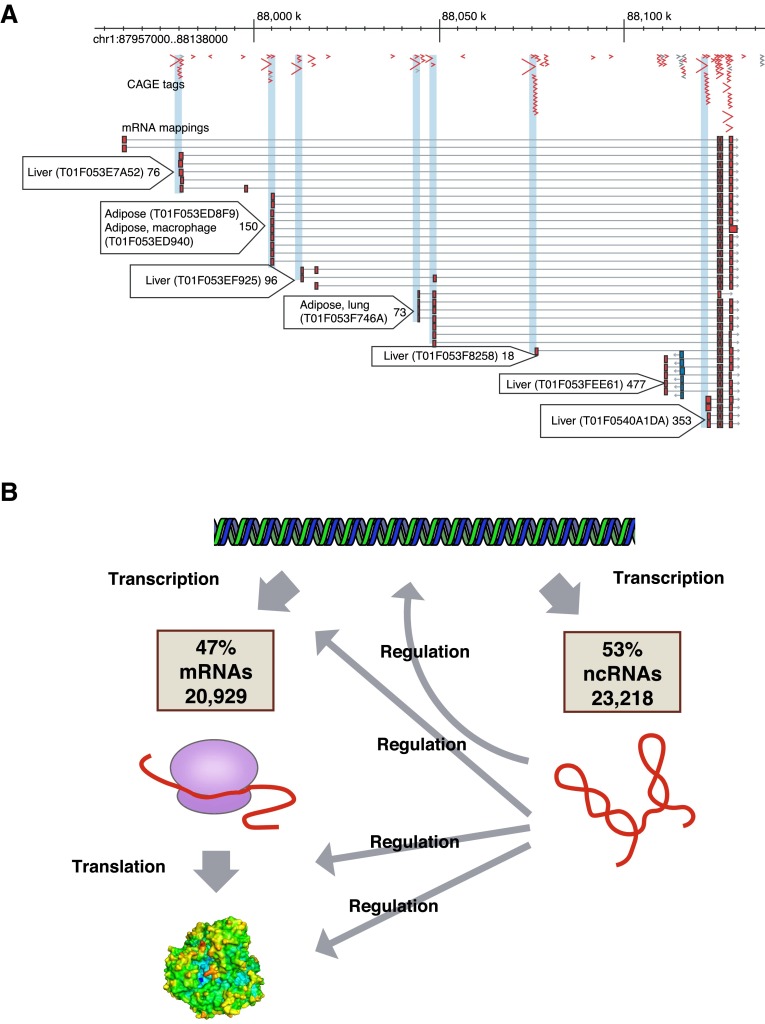
**a** An example of seven alternative promoters within the transcriptional unit of UDP-glucuronyl transferase gene. *Light blue boxes* indicate groups of CAGE tags transcribed from specific tissues. Reproduced, with permission, from the original publication (Carninci et al. 2005). **b** The FANTOM3 work revealed that roughly 47 % of the transcribed RNAs are messenger RNAs, encoding for proteins, while 53 % of the transcribed RNAs do not encode for proteins. Numerous reports since the discovery revealed that non-coding RNAs are functionally versatile and have shown to interact and/or regulate various processes including transcription, translation, and epigenetic modifications

Using CAGE, we started to produce new type of data, which were first analyzed in the summer of 2004. Remarkably, CAGE analysis not only identified promoters and quantified their activity and the expression of known RNAs (Fig. 1a), but also more importantly, in conjunction with the larger number of cDNAs, we realized that there were many more RNAs in the mammalian transcriptome than we previously thought (Fig. 1b). Complementing the CAGE analysis with a paired-end tag sequencing method (Wei et al. 2004), we discovered that the genome is pervasively transcribed—with more than 63 % of the genome producing transcripts (Carninci et al. 2005), and more than 73 % of the genes showing some form of antisense transcription (Katayama et al. 2005). The discovery of pervasive transcription was confirmed using an independent technology based on tiling arrays for the human genome (Cheng et al. 2005). Using CAGE, we produced the first comprehensive promoter map both for human and mouse (Carninci et al. 2006), which was accompanied by other publications providing an in-depth analysis of chains of genes that map continuously or overlapping on the genome and are often co-regulated (Engström et al. 2006). CAGE also enabled the discovery of different classes of promoter architectures, revealing a significant overrepresentation of TATA boxes in promoters showing sharp TSSs, which were predominantly associated with tissue-specific transcripts, whereas broad TSS regions were associated with CpG islands and often corresponded to RNAs transcribed across a wide range of cells and tissues (Carninci et al. 2006; Lenhard et al. 2012). Among other findings, CAGE additionally provided a map of very large transcripts (Furuno et al. 2006) and the first catalog of expressed pseudogenes (Frith et al. 2006).

## FANTOM4: Connecting the dots by a transcriptional regulatory network

**Fig. 2:**
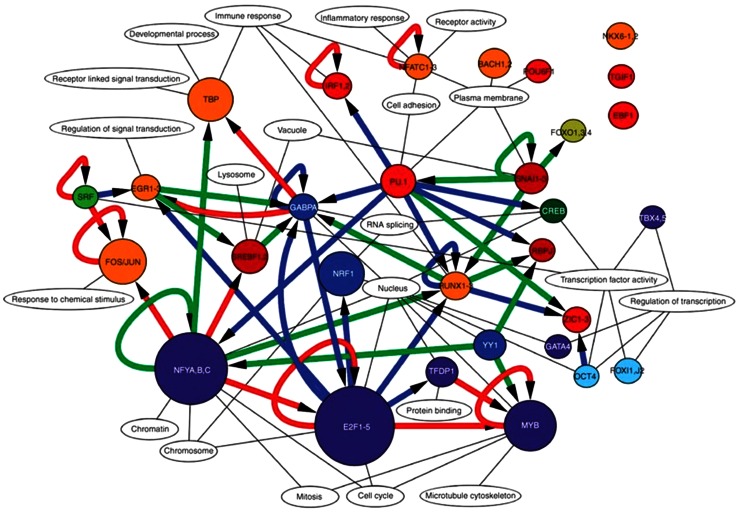
CAGE analysis in FANTOM4 revealed the concerted interplay of transcription factors during monocyte differentiation. Reproduced, with permission, from the original publication (Suzuki et al. 2009)

The appearance of next-generation sequencers (e.g., Roche-454) was instrumental in scaling up the production of CAGE libraries (Valen et al. 2009), which allowed sequencing of more than a million CAGE tags per sample, a depth that was sufficient to enable the analysis of gene networks. We centered the project on the dynamics of cellular differentiation of the THP-1 cell line from monoblasts to monocytes by CAGE profiling in a time-course experiment. The analysis of temporal CAGE data stimulated the development of a new set of bioinformatics tools that relied on the computational predictions of transcription factor-binding sites (Arnold et al. 2012), which are typically enriched at specific positions with respect to the TSS. The CAGE data guided the elucidation of the transcriptional network by providing the location of the exact TSS at single-nucleotide resolution and thereby pinpointing the genomic location where transcription factor-binding sites (TFBS) are most likely to be found. This study ultimately led to the publication of the first transcriptional regulatory networks based on CAGE analysis (Suzuki et al. 2009), revealing a remarkably complex interplay of transcription factors either activating or inhibiting each other (Fig. 2).

Unexpectedly, FANTOM4 generated several additional important findings. For example, the discovery of tiny RNAs (tiRNA) that map downstream of TSS; these RNAs may have a regulatory role, or alternatively, be a side product of transcription (Taft et al. 2009). Even more surprisingly, by considering tags derived from retrotransposon elements (REs)—which are usually discarded because of mapping challenges—we found that REs are frequently transcribed in a very cell type specific and reproducible manner (Faulkner et al. 2009). This provided evidence that REs contain specific promoters driving the transcription of coding and non-coding RNAs in various tissues, especially in embryonic stages where LINE and LTR elements are particularly active. The transcripts derived from REs were further studied in later projects, revealing their importance for stem cell maintenance (Fort et al. 2014; Kelley and Rinn 2012). This discovery underscores the importance of data-driven science in analyzing the experimental results, unbiased by preconceptions of how cells should behave according to the conventional biological knowledge: important conclusions may be missed if looking for the expected outcome only, and nature may surprise us by divulging its secrets in ways not anticipated when the experiment was originally designed.

## FANTOM5: Complete landscape of human and mouse promoterome

**Fig. 3:**
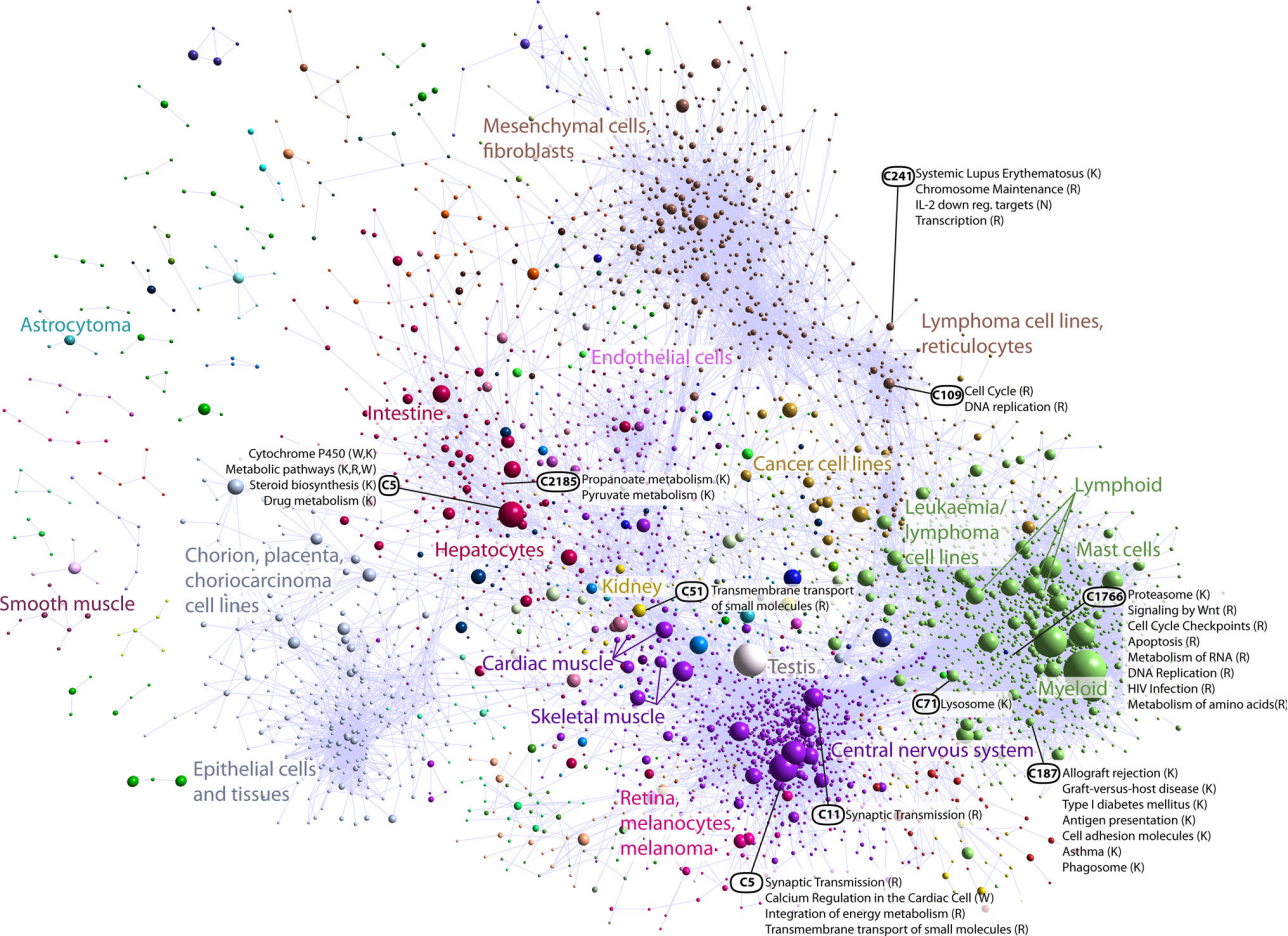
FANTOM5 profiled the broadest collection of human cell types using CAGE; the co-expression network shown here revealed clusters of cell types based on similar function and developmental lineages. Reproduced, with permission, from the original publication (Forrest et al. 2014)

Next-generation sequencing further shaped the CAGE technology (Kanamori-Katayama et al. 2011; Itoh et al. 2012; Takahashi et al. 2012), allowing the fifth edition of FANTOM to return to the comprehensiveness of the earlier FANTOM projects and to provide genome-wide transcriptome maps covering very broad sets of human cell types. FANTOM5 differed in two essential aspects from the ENCODE project (The ENCODE Project Consortium 2012), which was in progress around the same time. First, while ENCODE used immortalized cells in its analyses, FANTOM5 used a wide variety of human primary cells and tissues to capture the basic biology involved in establishing and maintaining cell type identity (Fig. 3). Second, while ENCODE encompassed many different technologies to probe the human transcriptome and epigenome, FANTOM5 largely used CAGE as a single readout of transcriptional activity, leveraging on data published in other projects for complementary analysis (for example, chromatin status in specific cell types); this allowed us to detect TSS and therefore the regulatory promoter regions both for coding and non-coding transcripts, and at the same time to quantify their transcriptional activity.

In the first phase of FANTOM5, we produced the largest mammalian transcriptome atlas based on CAGE profiling at single-nucleotide resolution (Forrest et al. 2014). This revealed that mammalian promoters typically contain multiple TSSs with different expression patterns across the samples in the expression atlas, implying that these TSSs, in spite of their proximity to each other, are regulated separately. Promoters expressed ubiquitously in different cell types showed the highest degree of conservation in the promoter sequence, while the sequences of cell-specific promoters tended to be less conserved.

As often happens, serendipity helps to discover novel biological principles in the data. Previously, it had been shown that enhancers are broadly marked by bidirectional transcription (Kim et al. 2010; Djebali et al. 2012). In the FANTOM5 data, however, we found that such enhancer-derived RNAs (mostly non-coding RNA, for which the function is still being debated) are expressed with a strong tissue and cell specificity reflecting the activity of enhancers. This led to the creation of a map of 44,000 active enhancers (Andersson et al. 2014). In contrast to identifying enhancers based on DNase hypersensitivity sites—which may also be associated with other biological functions—CAGE provided direct evidence for enhancer activity.

In the second phase of FANTOM5, we explored the dynamics of enhancers and promoters by CAGE profiling in 19 human and 14 mouse time-course experiments (Arner et al. 2015). While these time courses encompassed a wide diversity in cellular systems and included both their response to external stimuli as well as differentiation processes, in general, we found that the earliest response of cells in these time courses occurred at enhancers, followed shortly thereafter by the activation of the promoters of genes coding for transcription factors.

As in previous FANTOM projects, the fifth edition of FANTOM included a large number of satellite papers exploring specific aspects of the data in more detail. Integrating the FANTOM5 CAGE expression atlas with complementary data such as ChIP-Seq and DNA methylation profiling allowed us to study regulation in particular cell types or processes. This led to the discovery of the concerted action of transcription factors, enhancers, and DNA methylation during granulopoiesis (Rönnerblad et al. 2014), the landscape of promoters and enhancers of classical, intermediate, and non-classical monocytes (Schmidl et al. 2014), the interplay of DNA methylation and transcriptional regulation by Foxp3 in regulatory T-cells (Morikawa et al. 2014), and the role of bone morphogenetic protein receptors in the activation of mast cells (Motakis et al. 2014). On a global scale, analyzing the transcriptome output of human cells across the FANTOM5 compendium enabled us to understand the contribution of protein evolution to cell type identity (Sardar et al. 2014), as well as the essential role of promoter architecture in explaining the ubiquity of gene expression across human cell types (Hurst et al. 2014).

To facilitate the analysis of this large compendium of transcriptome data, we developed the Zenbu visualization system (Severin et al. 2014), allowing the fast browsing of large next-generation sequencing datasets, detailed inspection of expression levels of thousands of experiments at particular genomic loci, as well as data sharing for specific collaborations.

The unprecedented deep CAGE sequencing efforts in the FANTOM and ENCODE projects, together with other genome-wide profiling efforts, led to a revolution in our understanding of the mammalian transcriptome. In stark contrast to the earlier view of non-coding parts of the genome as transcriptional deserts composed of junk DNA, Djebali et al. (2012) showed that at least 75 % of the human genome can be transcribed. Likewise, the FANTOM projects revealed the existence of transcriptional forests of overlapping transcripts on both strands of the DNA (Carninci et al. 2005) instead of genes with well-defined genomic start and end positions. This raises the question of the definition of a gene, in particular as transcripts can overlap multiple genes (Djebali et al. 2012).

The complexity of the mammalian genome is further highlighted by the discovery of CAGE tags in unexpected regions, for example, across exon–exon boundaries. These CAGE tags were found to be due to recapping after RNA processing (Fejes-Toth et al. 2009); the biological function of these recapped transcript has not yet been determined. Integration of information obtained from CAGE with genome-wide data produced by complementary technologies is instrumental in interpreting the patterns observed in the data. For example, histone three acetylation profiling demonstrated that the exon-crossing CAGE tags are not due to independent transcriptional events (Fejes-Toth et al. 2009). Other examples include the combination of CAGE data with RNAseq experiments to connect transcription initiation events to novel transcript models (Djebali et al. 2012), as well as the confirmation of novel promoters identified using CAGE by H3K4 trimethylation marks and DNase hypersensitivity (Forrest et al. 2014), and CAGE-defined enhancers by H3K4 monomethylation and H3K27 acetylation (Andersson et al. 2014).

## FANTOM 6: Pioneering the world of lncRNAs

**Fig. 4:**
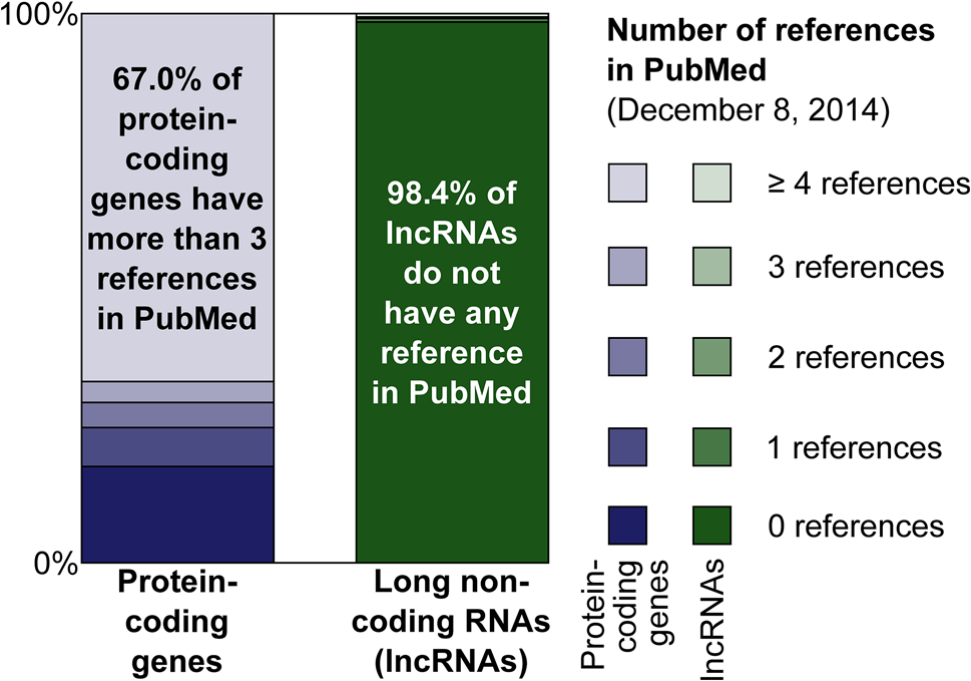
On December 8, 2014 we counted the number of references in PubMed for protein-coding and non-coding genes in release 21 of the GENCODE human gene set. Non-coding genes comprise of both lincRNAs and antisense transcripts as listed in GENCODE. We found multiple PubMed references for most protein-coding genes; in contrast, most non-coding RNAs did not have any reference in PubMed

While the FANTOM projects revealed a plethora of lncRNAs, the biological functions of these transcripts remain largely unknown. Currently, there is no reference in PubMed for more than 98 % of lncRNAs in Gencode (Harrow et al. 2012) (Fig. 4)—though this is a very conservative set of lncRNAs representing only a fraction of the tens of thousands of lncRNAs discovered in recent large-scale projects such as FANTOM, ENCODE (Djebali et al. 2012), and TCGA (Iyer et al. 2015), as well as FANTOM5 datasets yet to be published. At the same time, the lncRNAs that have been functionally characterized demonstrate that they have key roles across diverse biological processes, including the regulation of transcription, translation, the epigenome, and chromatin. For example, the lncRNA HOTAIR is involved in the genomic targeting of chromatin-modifying factors by scaffolding the assembly of ribonuclear proteins complexes (Rinn et al. 2007), suggesting that lncRNAs may be the adapters that drive the epigenome machinery to specific targets by providing a genomic zip code, yet to be decoded. More recently, we found an lncRNA transcript that regulates the level of translation of a protein-coding gene on the opposite strand of DNA; surprisingly, a SINEB2 repeat element transcribed as part of the lncRNA was found to be essential for its regulatory role in the cytoplasm (Carrieri et al. 2012). Such lncRNA were named SINEUPs, as they contain SINE elements that specifically UP-regulate translation. These lncRNAs be further artificially engineered to specifically enhance translation of targeted proteins (Patrucco et al. 2015; Zucchelli et al. 2015). These examples show that lncRNAs form direct interactions with proteins and complexes that are dynamic and can create specificity and control in regulation, and can thus be regarded as the master regulators of cellular function. Given that fundamental questions of biological regulation cannot yet be addressed by the protein-coding world, we believe that exploration of lncRNA function is essential to uncover regulatory elements.

However, attempts to comprehensively characterize the function of lncRNAs will be challenging. First of all, the definition of non-coding RNAs as transcripts that do not code for proteins is fundamentally based on what they are *not*, and it remains unknown whether this class of RNAs possesses any other encompassing characteristic. Long non-coding RNAs vary enormously in size: whereas lncRNAs are conventionally distinguished from short RNAs by having a length over 200 nucleotides (Kapranov et al. 2007; Wang and Chang 2011), very long intergenic non-coding RNAs (vlincRNAs) can span more than 1 MB (Furuno et al. 2006; St Laurent et al. 2013). In contrast to coding transcripts, which are predominantly cytosolic, non-coding RNA tends to be localized to the nucleus (Djebali et al. 2012), where the transcriptome is particular complex (Cheng et al. 2005, Kapranov et al. 2007, Fort et al. 2014). On average, lncRNAs have lower expression levels than protein-coding transcripts, with more than 80 % of lncRNAs detected in the ENCODE cell lines present at less than 1 copy per cell (Djebali et al. 2012). We note though that the expression varies by orders of magnitude levels between non-coding RNAs; as an example, the lncRNA MALAT1 was one of the most abundant RNAs across the FANTOM5 samples (Forrest et al. 2014). In addition, lncRNAs that appear lowly expressed overall may be highly expressed in particular cells, or be abundant in specific compartments of the nucleus.

In the case of protein-coding genes, we can often assign a tentative function to novel transcripts by analyzing the encoded amino-acid sequence either based on their global similarity to known proteins or by the identification of specific protein domains. In contrast, the primary sequence of nucleotides of lncRNAs is much less informative than the amino-acid sequence of protein-coding genes, preventing us in virtually all cases from assigning a function to novel non-coding transcripts. Additionally, lncRNAs are poorly conserved across organisms, at least at the primary nucleotide sequence level, suggesting that a comparative analysis to understand lncRNA function will not be trivial, and will need the development of novel comparative analysis methodologies specifically designed for investigating lncRNA functionality, as well as methods to explore three-dimensional folding similarities of RNAs.

Given these challenges, even the most fundamental questions regarding the function of most lncRNAs remain unanswered. As an example, the proportion of the lncRNAs that are empirically functional is currently a topic of intense debate. But also the general principles of lncRNA biology are unknown. For example, given that proteins can be classified into homologous groups with similar functions, can we likewise designate classes of lncRNAs based on their function, functional domains, mechanism, and mode of operation? If so, how many classes are there, what are their common functions or mechanisms, and how are they integrated in the global regulatory networks governing cellular behavior? Finally, do the unique physical properties of RNA allow lncRNAs to be functional in ways that proteins cannot? In particular, whereas proteins comprise multiple functional domains characterized by properly folded structures, lncRNAs typically combine folded functional domains with unstructured functional regions that may base-pair with other nucleic acids, including genomic DNA, and may thus provide ribonucleoprotein complexes exquisite target specificity in a manner fundamentally different from direct binding of proteins to DNA.

Answering these questions will require a global approach to interrogate a large collection of lncRNAs to substantiate their importance and to elucidate their role in the central regulation of the mammalian genome. Because of challenges associated with developing appropriate assays for yet-to-be described functions, we envision a FANTOM6 project based on high-throughput screening (HTS) of lncRNAs followed by *molecular phenotyping* by CAGE to assess lncRNA function in multiple cell types. Moreover, development and integration of novel technologies will undoubtedly be key to the success of this large-scale effort. In addition to HTS and CAGE, technologies to decipher the secondary structures of lncRNAs (e.g., Parallel analysis of RNA structure (PARS); Kertesz et al. 2010), their associations to chromatin and proteins (e.g., chromatin isolation by RNA purification (Chu et al. 2011) or Hi-C sequencing to map long-range interactions in the genome (Lieberman-Aiden et al. 2009)) will be instrumental in understanding the mechanistic insights into their mode of action. Overall, the functional screening and molecular phenotyping by CAGE will be essential to characterize lncRNA that is involved in various biological processes both in health and disease.

## Conclusions

The traditional approach of characterizing one gene function at a time has been inefficient and oftentimes incomplete, requiring the production of a large amount of data for a single project with little chances to be reused. Among other consortiums, FANTOM has strongly contributed for creating the necessary resources to broadly categorize genes and their regulatory relationships. For example, the FANTOM website (http://fantom.gsc.riken.jp) and the ZENBU Genome browser (http://fantom.gsc.riken.jp/zenbu; Severin et al. 2014) are visited frequently, with almost 10 million accesses between May 2014 and April 2015, and the publications derived from FANTOM have been cited 11,855 times since the creation of the consortium. We believe that four main factors have contributed to the success of the FANTOM projects. First, development of original technologies, such as full-length cDNA cloning and CAGE, has enabled the production of original datasets that were not previously available. Under the umbrella of a large research institute such as RIKEN, it has been feasible to develop a new generation of technologies without the pressure to publish in the short term, to which smaller research groups are often exposed. Second, the critical mass of RIKEN staff with a wide variety of expertise was needed to streamline the technology and data production before launching into large-scale production (e.g., Kawaji et al. 2014). Because of the immense scale of the FANTOM projects, we ensured to provide easy access to well-standardized technologies and data to many laboratories; this invitation has attracted excellent collaborators to further strength the consortium. Third, the scientific coordination of the FANTOM consortium with a wide range of expertise that went beyond the field of genomics was essential. In-depth and lively discussions with experts from various research backgrounds (e.g., immunology, neurobiology, developmental biology, stem cell biology, and also different types of bioinformatics and data analysis) have been instrumental in generating unique and novel interpretations of omics data; through this collective effort of the consortium, we were able to overcome new challenges and expand the minds of the scientific community as a whole. This scientific model further promoted consortium-centered landmark publications followed by a large number of specialized reports. Fourth, pioneering toward new biological aims has sustained the consortium over 15 years, from the shear collection of genomic elements in the earlier FANTOM projects to the analysis of gene regulation and function in later FANTOM projects. Developing key technologies to address evolving biological goals has thus been critical to the success of the consortium. We believe that further development of single cell methodologies and functional characterization of non-coding RNAs will play important roles in the future chapters of FANTOM. In conclusion, the FANTOM collaborative research model can be further implemented toward other biological aspects, ranging from elucidating cellular heterogeneity to understanding individual diversity in health and disease.
